# Discovering how ice crystals grow using neural ODEs and symbolic regression

**DOI:** 10.1126/sciadv.aee6174

**Published:** 2026-09-25

**Authors:** Kara D. Lamb, Jerry Y. Harrington, Alfred M. Moyle, Gwenore F. Pokrifka, Benjamin W. Clouser, Volker Ebert, Ottmar Möhler, Harald Saathoff

**Affiliations:** ^1^ Department of Earth and Environmental Engineering, Columbia University, New York, NY, USA.; ^2^ Learning the Earth with Artificial Intelligence and Physics Center, Columbia University, New York, NY, USA.; ^3^ Department of Meteorology, Pennsylvania State University, State College, PA, USA.; ^4^ High Meadows Environmental Institute, Princeton University, Princeton, NJ, USA.; ^5^ Department of the Geophysical Sciences, University of Chicago, Chicago, IL, USA.; ^6^ Physikalisch Chemisches Institut, Heidelberg University, Heidelberg, Germany.; ^7^ Physikalisch-Technische Bundesanstalt, Braunschweig, Germany.; ^8^ Reactive Flows and Diagnostics, Technische Universität Darmstadt, Darmstadt, Germany.; ^9^ Institute of Meteorology and Climate Research Atmospheric Aerosol Research, Karlsruhe Institute of Technology, Eggenstein-Leopoldshafen, Germany.

## Abstract

Depositional ice growth strongly influences the evolution and lifetime of ice-containing clouds on Earth and Mars, yet its physics remain uncertain because the earliest stages of ice-crystal growth cannot be directly observed and are sparsely sampled in laboratory settings. Here, we use physics-informed machine learning to learn the functional dependence of unknown physics in the depositional ice growth model from limited observations. By optimizing against mass growth time series from 290 ice crystals grown in a levitation diffusion chamber, we identify a modified capacitance growth model that more accurately captures observed early-stage growth. The resulting functional form, retrieved using symbolic regression, outperforms existing parameterizations and generalizes well to independent measurements from the AIDA Aerosol and Cloud Chamber. Our data-efficient learning framework provides an improved representation of depositional ice growth and offers a pathway to infer unresolved physical processes in other systems that are sparsely observed.

## INTRODUCTION

Cirrus clouds, pure ice clouds that form in the Earth’s upper troposphere, play a substantial role in the climate cycle (*1*). Mid-latitude and tropical cirrus cover approximately one-third of the upper troposphere at any point in time and have significant direct radiative climate effects. In addition to their radiative effects, the high, thin cirrus clouds that form in the Earth’s tropical tropopause layer act as an important control on the amount of water vapor that enters the stratosphere from the troposphere, where it is an important greenhouse gas (*2*). Contrail cirrus forming as the result of commercial aviation represents a significant but uncertain, anthropogenic climate forcing (*3*). Uncertainties in ice microphysical process rates that control cirrus cloud formation and lifetime translate to direct climate radiative forcing uncertainties on the order of 30 W/m^2^, more than eight times the radiative forcing associated with a doubling of CO_2_ in the atmosphere (*4*, *5*).

**Fig. 1.**
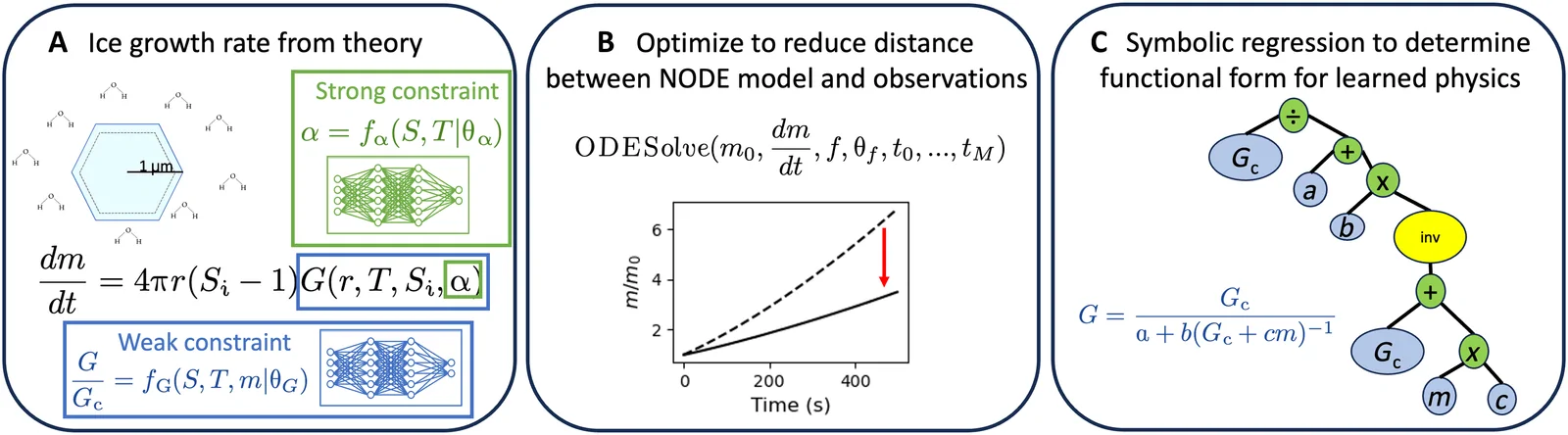
Overview of methodology for learning unknown physics in depositional ice growth models. Depositional ice growth is an important process for ice formation in atmospheric clouds. Ice crystals grow via direct deposition of water molecules from the vapor phase onto the ice surface. (**A**) We replace partially unknown physics in the depositional ice growth model with a neural network, considering both a strong and weak constraint. (**B**) We integrate the ice growth rate partially parameterized by a neural network and optimize to reduce the distance between the model and experimentally measured time series of ice mass ratios. (**C**) We use symbolic regression to determine a functional form for the unknown physics learned by the neural network.

One of the most important microphysical processes controlling the growth and evolution of cirrus clouds is the depositional ice growth rate, which describes the adsorption, surface migration, and incorporation of water molecules into the ice crystal (Fig. 1A). However, substantial gaps in our understanding of depositional ice growth, particularly the early growth of ice crystals in clouds, continue to limit our ability to accurately model these processes (*4*, *6*). Early growth is especially hard to constrain because this growth connects freshly nucleated particles to larger faceted crystals. These small ice crystals (radii of <50 μm) undergo various surface transformations as habit forms develop, affecting the growth rates (*7*–*10*).

Structural and parametric uncertainty in ice growth models has been difficult to address (*11*). Structural uncertainty refers to uncertainty in the functional dependence of a physical model, whereas parametric uncertainty refers to uncertainty in parameter values. While depositional ice growth is typically represented in cloud and climate models with capacitance theory (*12*), the early stages of ice growth leading to the formation and growth of facets is determined by surface attachment kinetics, which are poorly constrained by current theories, observations (those made in clouds), and laboratory measurements. Measurements of surface attachment kinetics modeled as a deposition coefficient from different experimental studies disagree by orders of magnitude (*13*, *14*), although recent work has suggested these studies can be reconciled with a saturation and temperature dependent functional form (*15*, *16*). One major challenge in constraining depositional ice growth rates in laboratory studies is that the early stages of growth cannot be directly observed and must instead be inferred from observables such as the mass and often in conditions where one or more dependent variables (such as temperature or supersaturation with respect to ice) are held constant.

Here, we use physics-informed machine learning to address structural uncertainty in the depositional ice growth model. Scientific machine learning for discovering unknown physics directly from observations has demonstrated substantial promise in recent years (*17*–*19*). Approaches such as physics-informed neural networks (*20*) can be leveraged to integrate observational data with known governing physical laws, even in cases with partially unknown physics. Neural ordinary differential equations (NODEs) integrate neural networks parameterizing an unknown function with typical numerical ordinary differential equation (ODE) solvers to learn a continuous model for a physical system (*21*). In this study, we develop a methodology to learn the functional dependence for the ODE describing the depositional ice growth rate by optimizing a NODE across multiple time series simultaneously. We compare strongly constrained and weakly constrained NODE models, based on the amount of prior physical knowledge that is included. We apply this method to experimental measurements to learn a mathematical model for the depositional ice growth rate and use symbolic regression to discover closed form expressions for the nonlinear relationship learned by the neural network. Last, we use an independent dataset of cirrus cloud experiments performed in the AIDA (Aerosol Interaction and Dynamics in the Atmosphere) Aerosol and Cloud Chamber to select among candidate proposed ice growth models.

### Levitation diffusion chamber observations

Levitation diffusion chamber experiments provide time-resolved measurements of individual ice crystals growing in a highly controlled environment. Here, we explore how scientific machine learning and equation discovery can be applied to measurements of both heterogeneously and homogeneously nucleated ice crystals grown in a levitation diffusion chamber at temperatures between 205 and 240 K and saturation with respect to ice between 1.0 and 1.8, conditions characteristic of upper tropospheric cirrus cloud formation (*9*, *22*, *23*). During experiments, individual ice crystals with initial radii between 6 and 26 μm are nucleated, levitated, and grown from vapor in constant saturation $S_{i}$ and temperature *T* conditions. Measurements consist of 290 time series of the mass ratio ($m/m_{0}$) of individual ice crystals, where *m* is the mass of an ice crystal and $m_{0}$ is its initial mass. The crystals remain relatively small over the course of the experiment, with equivalent spherical radii typically <40 μm (maximum of ∼84 μm). Because mass ratio time series have varying durations (depending on how efficiently the ice crystals grow), we interpolate time series to 1 Hz and crop all datasets to a maximum length of 500 s. The $S_{i}$, *T*, and mass time series for all 290 experiments used in the analysis are shown in fig. S1.

In addition to the experimentally measured ice crystal mass ratio time series, we create a synthetic dataset of levitation diffusion chamber measurements with a known functional dependence for the depositional growth rate to validate the performance of our equation discovery method. This synthetic dataset consists of 290 mass ratios of ice crystals growing in the same saturation $S_{i}$ and temperature *T* conditions and with the same initial mass $m_{0}$, but with an assumed functional form for the depositional ice growth model based on (*24*) (fig. S2). Realistic measurement noise is added to the synthetic time series by using the trailing eigenmodes of a singular spectrum analysis (SSA) decomposition applied to the measured time series (fig. S3 shows an example). Additional details about experimental and synthetic datasets are given in Materials and Methods.

### Methodology to learn unknown physics

In this section, we describe the scientific machine learning methodology that we use to discover a mathematical model for depositional ice growth that best fits all 290 levitation diffusion chamber experiments. An overview of the method is shown in Fig. 1. The depositional ice growth rate for an ice crystal growing from vapor has traditionally been modeled using the capacitance ice growth model (*12*), which is modified to include surface kinetics for the early stages of ice growth (*15*). This model is an ODE

$$\frac{\mathrm{dm}}{\mathrm{dt}}=4\pi r\left(S_{i}-1\right)G\left(r,T,S_{i},\alpha \right)$$ (1)

where *m* is the mass of the crystal, *r* is the radius of the ice crystal, and $S_{i}$ is the ambient ice supersaturation. The function $G\left(r,T,S_{i},\alpha \right)$ represents the combined effects of gas-phase vapor and thermal diffusion along with surface attachment kinetics typically parameterized by a deposition coefficient α. Here, we assume that the capacitance *C* scales proportionally with *r*, consistent with the standard approximation for spherical ice particles. As a result, the transfer coefficient *G* also incorporates unresolved physical effects associated with the evolving morphology of ice crystals during the early stages of growth. The deposition coefficient α has previously been parameterized as a saturation and temperature-dependent function in (*24*) (Eq. 10). However, this form may not be valid for crystals undergoing rapid transformations immediately following nucleation (*8*). A more detailed description of the capacitance ice growth model with modifications for surface kinetics is provided in Materials and Methods.

Predicting ice growth in the levitation diffusion chamber amounts to solving an initial value problem, where the ice crystal mass as a function of time is given by

$$m\left(t\right)=\int_{0}^{t}\frac{\mathrm{dm}}{\mathrm{dt}}\mathrm{dt}+m_{0}$$ (2)

If we knew the functional dependence of Eq. 1, then this problem would be straightforward to solve. However, the functional dependence for $G\left(r,T,S_{i},\alpha \right)$ is unknown. This uncertainty arises from the difficulty in consistently parameterizing various surface transformations that ice crystals undergo in their early stages of growth and the lack of observational constraints on the evolving habit of these micron-scale ice crystals. While prior attempts to parameterize size-based surface kinetics due to transformations have been made, most have been either ad hoc (*25*) or lack generality due to the use of smaller datasets (*26*). In addition, because each ice crystal grows at a constant, but unique $S_{i}$ and *T*, determining a functional dependence of $G\left(r,T,S_{i},\alpha \right)$ on *r*, *T*, and $S_{i}$, and with a possibly saturation and temperature-dependent α that is consistent across all experiments is not straightforward.

Here, we focus on learning a single depositional ice growth model that best fits all experiments, with the aim of developing a simple parameterization for cirrus cloud models. We use a NODE model to solve Eq. 2 to learn a consistent functional form for $G\left(r,T,S_{i},\alpha \right)$ across all 290 experiments simultaneously. A NODE model naturally formulates the problem as learning a dynamical growth law from observed ice crystal growth trajectories while avoiding the need to estimate noisy time derivatives from experimental measurements. We assume two cases for prior physical knowledge in Eq. 1 based on past literature (Fig. 1A). The first model makes a stronger assumption about the amount of prior physical knowledge to include, whereas the second model learns a greater part of the ice growth model from experimental measurements. In the first case, we use a capacitance model for ice growth that assumes an unknown function for the deposition coefficient α, which we refer to as the “strongly constrained NODE” model. In the second case, we fit the ratio of the transfer coefficient *G* relative to the theoretical transfer coefficient $G_{c}$ for a spherical ice crystal assuming the continuum limit (Eq. 12), which we refer to as the “weakly constrained NODE” model. In each case, we use prior physical knowledge from Eq. 1 and replace only the uncertain portion of the model with a neural network. For the strongly constrained NODE model, we assume the functional form for $G\left(r,T,S_{i},\alpha \right)$ is given by Eq. 8, and we further assume that α is a function of temperature and supersaturation

$$\alpha =f_{\alpha }\left(S_{i},T|\theta_{\alpha }\right)$$ (3)

The expression $f_{\alpha }\left(S_{i},T|\theta_{\alpha }\right)$ represents a neural network that takes as input $S_{i}$ and *T* and given the weights of the neural network θ_α_ predicts a value for α. For the weakly constrained NODE model, we assume that the ratio between *G* and $G_{c}$ is given by a function of the temperature, supersaturation, and mass of the ice crystal

$$\frac{G}{G_{c}}=f_{G}\left(S_{i},T,m|\theta_{G}\right)$$ (4)

Parameterizing kinetics with a modified *G* is advantageous because it is more general, and it has some measurement (*9*) and theoretical (*27*) backing.

To compare against experimental measurements (Fig. 1B), we integrate Eq. 1 partially parameterized by neural networks using an ODE solver [implemented in PyTorch with the torchdiffeq library; (*21*)]. We optimize the strongly and weakly constrained NODE models against the experimental time series to simultaneously learn the neural network weights θ_α_ and θ_G_ in Eqs. 3 and 4, respectively, that reduce the distance between the models and the measured time series. Because we assume that all 290 experimental mass time series can be modeled with the same physical model, we optimize the NODE models across all time series simultaneously by minimizing L2 loss [or mean square error (MSE) loss] between the measured time series and the model

$$L_{\text{NOD}E}=\sum_{j=0}^{N_{\text{exp}}}\sum_{i=0}^{T_{j}}{\left[\frac{m_{j}\left(t\right)}{m_{j,0}}-\frac{{\hat{m}}_{j}\left(t\right)}{m_{j,0}}\right]}^{2}$$ (5)

where *j* is the experiment number, $T_{j}$ is the length of the *j*th time series, ${\hat{m}}_{j}\left(t\right)$ indicates the prediction from the NODE models, and $N_{\text{exp}}=290$.

Once the NODE models have been optimized against experimental measurements, we use symbolic regression to derive functional forms for the trained neural networks (Fig. 1C). Symbolic regression uses genetic algorithms to search for equations that optimize accuracy while limiting model complexity. Here, we use the symbolic regression library PySR to return a Pareto front of candidate expressions (*28*). We use the observed values for $S_{i}$ and *T* and the initial mass $m_{0}$ for each of the 290 experiments. These values are used as input for the trained neural networks to determine the corresponding values for α for the strongly constrained NODE model (Eq. 3) and for *G* for the weakly constrained NODE model (Eq. 4). Further details of the strongly and weakly constrained NODE models, the training process, and the equation discovery method are given in Materials and Methods.

## RESULTS

**Fig. 2.**
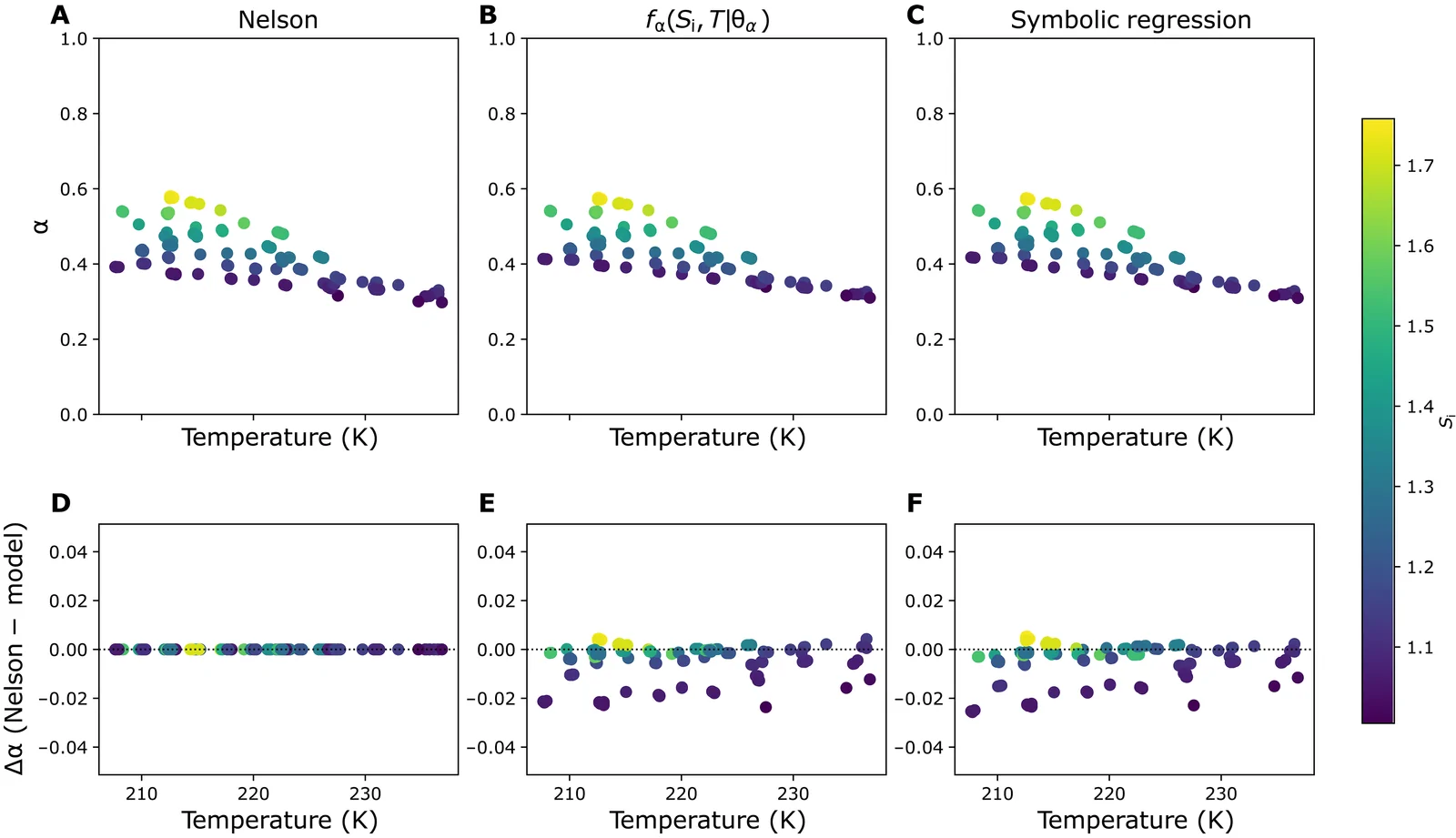
Functional dependence of α learned from synthetic datasets. (**A**) Saturation and temperature dependence of α parameterization from Nelson and Baker (*24*). (**B**) Saturation and temperature dependence from the trained NN for the synthetic datasets. (**C**) Predictions from an expression learned by symbolic regression from the trained NN for the synthetic datasets. (**D** to **F**) Residuals between α from Nelson and Baker (*24*) and model predictions for α.

We first test our method on the synthetic datasets with a known functional form for the deposition coefficient α based on (*24*). After optimizing the strongly constrained NODE model against the synthetic datasets, we find that the model is able to reproduce the time series very accurately (fig. S4 shows examples of a subset of time series compared with NODE model fits). We compare the predicted values of α from the trained neural network α_NN_ to those used to generate the synthetic dataset α_Nelson_. The strongly constrained NODE model is able to learn the nonlinear functional dependence for α (Eq. 10) that closely matches the one used to generate the synthetic datasets (Fig. 2, left and middle). In addition, when we use symbolic regression to learn a functional dependence for α from the trained neural network, it closely matches the true dependence (Fig. 2, right).

We next use our approach on the experimentally measured time series. Because we have no ground truth for the depositional ice growth model for the experimental datasets, we compare the performance of the weakly and strongly constrained NODE models optimized against the experimental datasets with the model from Nelson and Baker (*24*) and a model with no surface kinetics (Table 1). We evaluate the MSE loss between models and experimental measurements across all 290 experiments (Eq. 5), as well as evaluating which model performs best on the majority of individual experiments (determined by the model which gives the lowest MSE loss for an individual experiment). According to both metrics, the weakly constrained NODE model outperforms a model with no surface kinetics, the Nelson and Baker (1996) model and the strongly constrained NODE model. The weakly constrained NODE model performs best on 138 of the 290 experiments (Table 1, third column). When considering performance on individual experiments, the strongly constrained NODE model performs better than the Nelson and Baker (1996) parameterization, but only slightly better than a model with no surface kinetics.

**Table 1.** Comparison of model performance on experimental data from the levitation diffusion chamber. MSE loss between model and experiments is evaluated across all 290 time series; we separately evaluate the interpolation performance (*t* < 500 s) and the extrapolation performance (*t* < 1000 s). Best experiment count refers to the total number of individual experiments for which the proposed model performed best. The bold entries indicate the best performing model according to the given metric.

Ice growth model	MSE loss	MSE loss	Best exp. count	Best exp. count
	(*t* < 500 s)	(*t* < 1,000 s)	(*t* < 500 s)	(*t* < 1,000 s)
No surface kinetics (*G* = $G_{c}$)	41,396	222,750	62	55
Nelson and Baker (*24*)	40,751	220,930	29	31
Strongly constrained NODE model	30,714	203,645	61	62
Weakly constrained NODE model	**16,495**	**184,723**	**138**	**142**

**Fig. 3.**
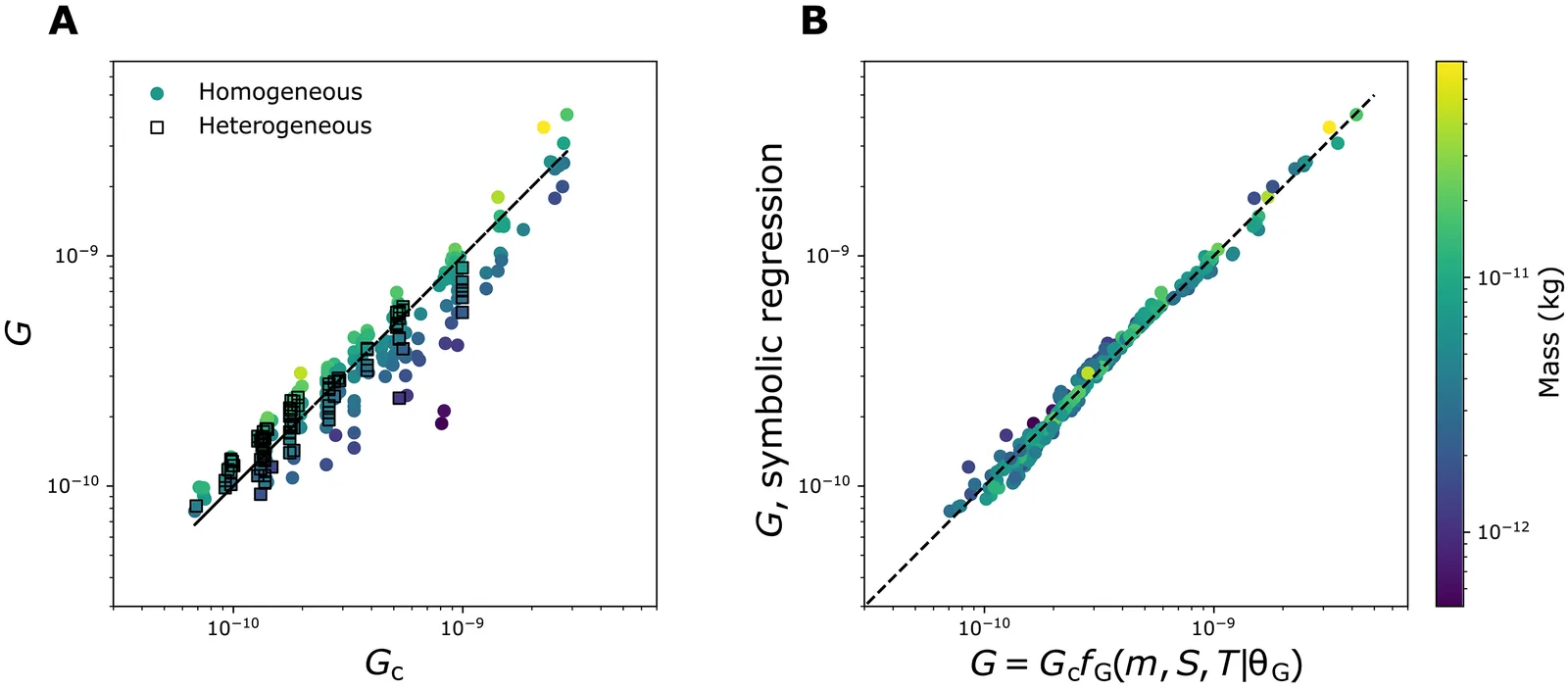
Unknown physics learned by weakly constrained NODE model from experiments. (**A**) The transfer coefficient *G* learned by the weakly constrained NODE model for the 290 experiments compared with $G_{c}$ (the transfer coefficient for the continuum case, assuming a spherical ice crystal). (**B**) Predictions from the trained neural network for *G* compared with the predictions from an expression learned by symbolic regression from the trained neural networks (eq. 8 in table S2) for the 290 experiments.

In comparing the strongly constrained NODE model optimized against the experimental datasets with the measured mass ratios, we find substantial deviations between the measured mass ratios and predicted mass ratios for some experiments (fig. S5). The predicted values of α from the trained neural network also differ substantially from those predicted by the functional form given in (*24*) and suggest that the neural network struggles to find a consistent smoothly varying function across $S_{i}$ and *T* (fig. S6). As the strongly constrained NODE model is not able to learn a functional dependence for α that reproduces the mass ratio time series for the majority of the experiments, this suggests that a model using a single deposition coefficient form α may not be the optimal functional relationship to describe the depositional ice growth process for newly formed crystals. By contrast, when we optimize the weakly constrained NODE model against the experimental datasets, this generally reduces deviations between the mass ratio time series and the predicted values for the mass ratios (fig. S5) compared with the strongly constrained NODE model. In addition, we can use the trained neural network to evaluate the learned functional dependence of *G* compared with $G_{c}$ (Fig. 3A), and our model learns a consistent functional dependence across the 290 experiments. While *G* is generally within a factor of 1.2 of $G_{c}$ for most experiments, the learned functional dependence indicates that there is an additional dependence on *m* that is not accounted for by $G_{c}$.

As described in the previous section and in Materials and Methods, we use symbolic regression to determine a functional dependence for *G* learned by the neural network. Symbolic regression discovers multiple candidate expressions for *G* (see table S2). All discovered expressions depend on $G_{c}$ (as given by given by Eq. 12) and *m*, the crystal mass. Only the most complex expressions show any dependence on $S_{i}$ and *T*, indicating that the majority of the variance in the physical model can be related to the ice crystal size. This functional dependence is consistent with physical expectations for surface kinetic effects, which should suppress ice growth when ice crystals are small and habits are just forming. More complex expressions typically have lower MSE loss (fig. S7), and we further evaluate these expressions on independent data (discussed in the next section) to determine which equation provides the optimal balance between complexity and generalization performance.

### Performance of proposed models on unseen data

To select among the learned depositional ice growth models, we evaluate our proposed models for depositional ice growth on data that was unseen during the training process of the NODE model.

Because we only used the initial 500 s from each time series for the ice crystals grown in the levitation diffusion chamber, we first test how well models perform on predicting the mass ratio of the ice crystals for the remainder of the experimental time series (e.g., for *t* > 500 s). However, we do note that this extrapolation test will necessarily be biased toward ice crystals that are compact and grow less efficiently, typically at lower supersaturations. High supersaturation promotes polycrystalline growth, which is rapid, and the time series measurements for such crystals are relatively short and thus may not be represented in this dataset. We test how well the models perform by integrating the time series up to 1000 s and evaluating the MSE loss between the measured time series and the model (Table 1, second column). In this case, the weakly constrained NODE model still performs best, but the performance is more comparable with the other three proposed models. In addition, when integrating for longer times greater than 1000 s, the weakly constrained NODE model begins to perform worse relative to the other three models as the weakly constrained NODE model begins to overpredict ice growth at larger sizes, which is consistent with longer time series measurements being associated with less efficient growth. This suggests that the weakly constrained NODE model is an improvement on the previous models when *m* is small, but that in the limit where *m* is larger, depositional ice growth should asymptote to the continuum limit.

Next, we evaluate how well the proposed depositional ice growth models can predict ice growth in a completely independent dataset, using observations from adiabatic expansion experiments simulating cirrus cloud formation in the AIDA Aerosol and Cloud Chamber during the IsoCloud campaign (*16*, *29*, *30*). These experiments heterogeneously and homogeneously nucleated populations of ice crystals, which then grow in conditions characteristic of upper tropospheric cirrus cloud formation over ∼10 min. The $S_{i}$ and *T* range for the AIDA experiments overlap with the levitation diffusion chamber experiments, and also includes experiments at lower *T* and higher $S_{i}$ and time-evolving (rather than fixed) environmental conditions (see fig. S8). In AIDA, we observe the total mass of the population of ice crystals in a volume of air [the ice water content (IWC)] as a function of time, rather than the mass of individual ice crystals. To compare our proposed depositional ice growth models against the AIDA experiments, we model ice growth in AIDA using a parcel model with bin microphysics constrained to the observed number concentrations of ice, as was previously described in (*16*). We replace the depositional ice growth model with the learned expressions from symbolic regression for *G* given in table S2 and use the AIDA experiments to select among proposed functional forms for *G*, by determining which model best fits the majority of AIDA experiments. We find that the following functional form (discovered eq. 8 in table S2)

$$G=a_{0}G_{c}^{a_{1}}{\left[a_{2}+\frac{a_{3}}{m}\right]}^{-1}+a_{4}$$ (6)

where $a_{0}=688.267$, $a_{1}=1.3153$, $a_{2}=0.85601$, $a_{3}=2.6606\times 10^{-12}$, and $a_{4}=0.1123\times 10^{-9}$, improves upon the capacitance ice growth (e.g., no surface kinetics) and the Nelson and Baker (1996) models at lower temperatures in AIDA (fig. S9). Some of the residual disagreement between all ice growth models and the AIDA observations at low temperatures (<200 K) likely reflects systematic measurement uncertainty rather than deficiencies in the growth models themselves (*16*, *31*–*33*). We note that this discovered functional form for *G* has a mass dependence that is reminiscent of some earlier depositional ice growth theories that approximate attachment kinetics with a “kinetic length scale” (*27*, *34*), with $a_{3}$ playing the role of a kinetic length scale suitable for small cirrus-like ice crystals.

**Fig. 4.**
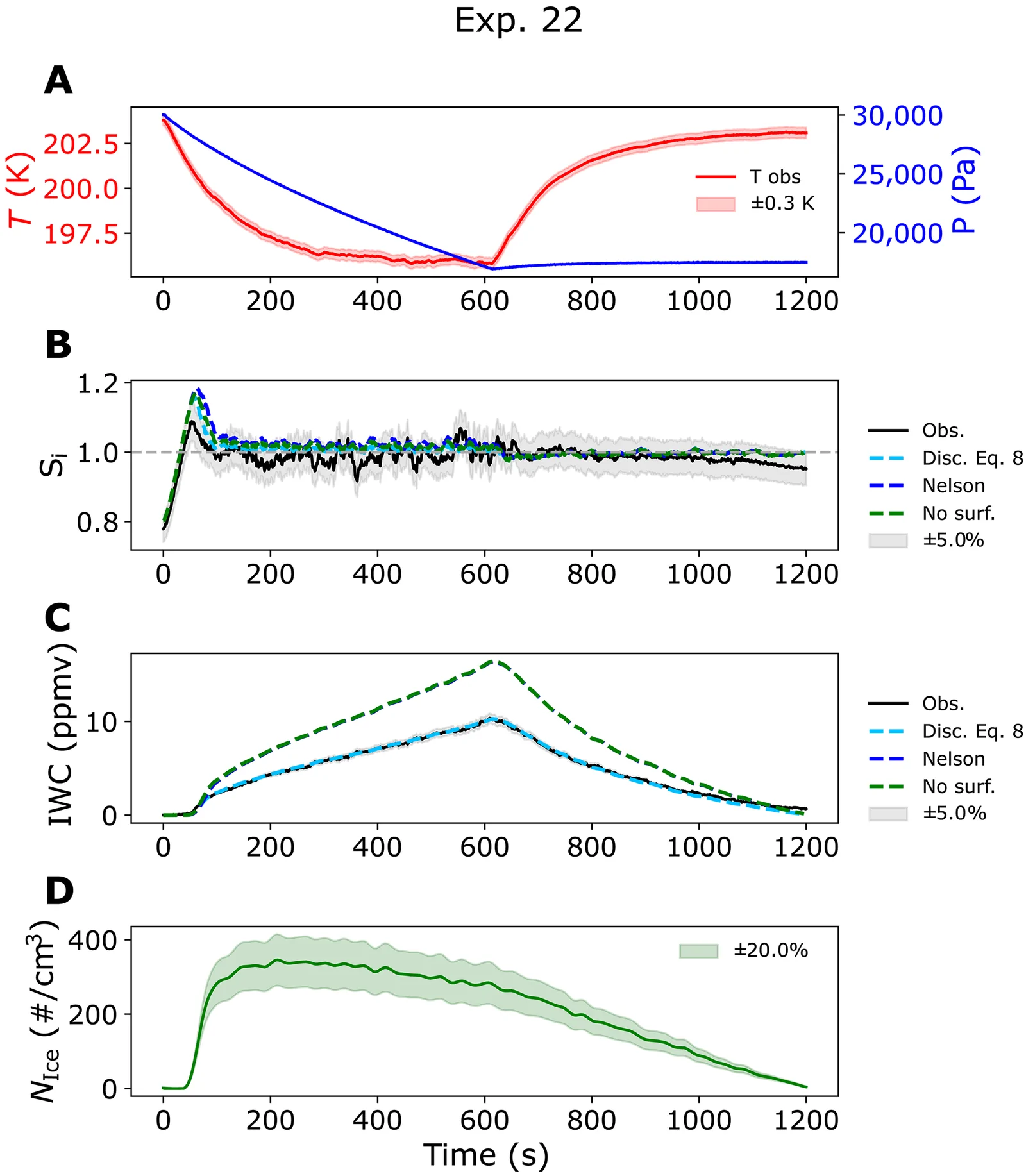
Comparison of learned depositional ice growth model to a cold cirrus cloud experiment in the AIDA Cloud Chamber. (**A**) Temperature and pressure during the adiabatic expansion experiment. (**B**) Observed supersaturation with respect to ice inside the chamber, compared with predicted supersaturation with respect to ice. Gray dashed line indicates supersaturation equal to 1.0. (**C**) Observed ice water content (IWC) compared with predicted IWC. (**D**) Observed ice number density inside of the chamber.

Figure 4 shows an example AIDA experiment modeled with the proposed model for depositional ice growth using Eq. 6, demonstrating that both supersaturation with respect to ice and the IWC over the course of the experiment are well predicted by our proposed model for depositional ice growth. The proposed model better predicts both the supersaturation with respect to ice (during the early stages of the experiment) and the evolving IWC than both the Nelson and Baker (1996) model and a model with no surface kinetic effects. The ability of our proposed model for depositional ice growth learned from the levitation diffusion chamber experiments to reproduce ice growth in the AIDA Cloud Chamber indicates that this model generalizes well to realistic conditions for cirrus cloud evolution.

## DISCUSSION

We have demonstrated the application of NODEs and symbolic regression to learn a mathematical model directly from multiple experimental time series of growing ice crystals. We validate our approach to learning the unknown part of a partially known ODE using a synthetic dataset with a known functional dependence for the deposition coefficient α. We find a weakly constrained NODE model is able to more accurately reproduce the majority of our experimental time series for the growth of newly formed crystals, suggesting that prior parameterizations for surface kinetic effects as a single deposition coefficient function may be too restrictive. This conclusion is consistent with (*9*) who found that newly formed crystals required transformations in the surface growth modes that control surface attachment kinetics. We instead propose a functional dependence for *G* in Eq. 1 that more accurately reproduces the experimental observations and that can be expressed as a closed-form function using symbolic regression (Eq. 6). This function also generalized well to unseen data, both when predicting longer ice growth time series in the levitation diffusion chamber and when modeling observations of populations of ice crystals growing in the AIDA Cloud Chamber.

This research contributes to the development of improved model parameterizations for cirrus cloud processes. Processes related to ice formation in clouds are a substantial source of uncertainty in current climate models (*11*, *35*). Detailed laboratory experiments are required to constrain individual microphysical process rates, but observables in laboratory experiments are often indirectly related to the prognostic variables used in atmospheric models. Here, we have focused on learning a single functional form that best fits all ice crystals; an alternative approach is to derive a distribution of growth rates from these experimental measurements (*23*). In addition to improving our fundamental understanding of the depositional ice growth process, the methods that we have introduced here can be more broadly applied toward model parameterization development for atmospheric and oceanic processes (*36*).

## MATERIALS AND METHODS

### Depositional ice growth model

The depositional ice growth rate for a single ice crystal growing from vapor (Fig. 1A) in the atmosphere has traditionally been modeled using the capacitance ice growth model, which describes the growth of an ice crystal from the vapor phase (*12*). The capacitance growth model, including modifications for surface kinetic effects, can be written as an ODE

$$\frac{\mathrm{dm}}{\mathrm{dt}}=4\pi C\left(S_{i}-1\right)G\left(r,T,S_{i},\alpha \right)$$ (7)

where *C* is the capacitance, $S_{i}$ is the ice supersaturation far from the crystal, and *G* represents the combined effects of vapor and thermal diffusivity to the surface of the ice crystal. *G* is a function of temperature, pressure, and the modified diffusivity $D_{v}^{*}$. The capacitance is a function that depends on the geometry of the ice crystal; here, we assume that C=r.

In the transitional regime between the continuum and the molecular limits, as is typically assumed for the early stages of atmospheric ice growth, the function *G* given in (*37*) is

$$G={\left[\frac{RT_{\infty }}{e_{\text{sat},i}\left(T_{\infty }\right)D_{v}^{*}M_{w}}+\frac{L_{s}}{k_{a}T_{\infty }}\left(\frac{L_{s}M_{w}}{RT_{\infty }}-1\right)\right]}^{-1}$$ (8)

Due to surface attachment effects, the diffusivity in Eq. 8 is modified from the continuum case. The modified diffusivity is defined below and includes influences of surface processes typically parameterized by a deposition coefficient α. Surface attachment kinetics refer to the combined effects of individual vapor molecules adsorbing, migrating, and attaching to surface sites on a growing crystal. These collective processes lead to effective differences in vapor diffusivity relative to the continuum case (where surface attachment effects are ignored). Unlike the accommodation coefficient for liquid droplets, which is often assumed to be near unity, the deposition coefficient for ice crystals represents aggregate surface kinetic and morphology-dependent limitations to molecular incorporation into the crystalline lattice. Note that kinetic influences on thermal conduction are generally small and are ignored here (*15*)

Here, we use the “modified” vapor diffusivity assuming spherical symmetry, which can expressed as

$$D_{v}^{*}=\frac{D_{v}}{\frac{r}{r+\Delta_{r}}+\frac{4D_{v}}{r\alpha w}}\;\text{where}\;w={\left(\frac{8{\mathrm{RT}}_{a}}{\pi M_{w}}\right)}^{1/2}$$ (9)

where $D_{v}$ is the diffusivity of water molecules in air, *r* is the radius of the ice crystal, *w* is the molecular speed of water vapor in air, $M_{w}$ is the molecular weight of water, *R* is the universal gas constant, and $T_{a}$ is the air temperature, $\Delta_{r}$ is the molecular jump distance, and α is the deposition coefficient.

The deposition coefficient α in Eq. 9 has previously been parameterized as a saturation and temperature dependent function in (*24*) as

$$\alpha ={\left(\frac{s_{\text{local}}}{s_{\text{crit}}}\right)}^{m}\text{tanh}\left[{\left(\frac{s_{\text{crit}}}{s_{\text{local}}}\right)}^{m}\right]$$ (10)

where $s_{\text{local}}$ is the supersaturation immediately above the crystal surface, $s_{\text{crit}}$ is the temperature-dependent critical supersaturation, and *m* is a parameter that relates to the surface of the ice crystals. A value of *m* = 1 is used to represent spiral dislocation growth, whereas a value of m>10 represents ledge nucleation. This parameterization is used to represent complex surface processes that are not fully represented by current theory. Here, we use the temperature dependent parameterization for $s_{\text{crit}}$ from (*15*) given by

$$s_{\text{crit}}=9.6066\times 10^{-5}T_{c}^{1.9171}$$ (11)

where $T_{c}$ represents the temperature in degrees Celsius.

In the continuum limit for a spherical ice crystal, α → 1 and Eq. 8 reduces to

$$G_{c}=G\left(D_{v}^{*}\to D_{v}\right)$$ (12)

Equation 7 relies on a number of constants and empirical expressions, which we summarize in table S1 and the Supplementary Text.

### Levitation diffusion chamber datasets

Experimental data were taken in the button-electrode levitation thermal gradient diffusion chamber and were described in detail in (*9*, *22*, *23*). Charged droplets are initially levitated between two ice-coated parallel plates, with the bottom plate having an opposing direct current voltage and the top plate an alternating current to stabilize particles horizontally. Due to differences in temperature between the warmer, upper plate and the colder, lower plate, a supersaturation gradient exists in the chamber as a function of height. After the ice crystal nucleates, the voltage of the bottom plate is automatically adjusted to maintain constant levitation of the ice crystal, with the measured voltage being directly proportional to the ice crystal’s mass as a function of time. The initial radius of the particle $r_{0}$ can be estimated from Mie theory using the diffraction pattern generated from the scattering of light from a Helium Neon laser, thus determining the initial crystal mass $m_{0}$. Deriving the mass as a function of time from the voltage measurement requires the assumption that ice is initially spherical, when it is likely poly-crystalline when it is initially nucleated (*38*). Because the uncertainty in derived mass is largely dominated by uncertainty in the initial size of the particle (*22*), we optimize the NODE model against $m/m_{0}$ rather than *m*.

Time series of the voltage of the lower plate are recorded at a 1-Hz frequency during the course of the experiments. Datasets consist of the measured temperature, pressure, supersaturation, initial ice particle radius observed from light scattering, and the voltage of the lower plate (proportional to the ice particle mass). Temperature uncertainty in the chamber is on the order of <1% (*22*). Supersaturation uncertainty is estimated to be on the order of 10% (*9*). Because supersaturation uncertainty is due to the location of the ice crystal in the chamber, it is likely to show a bias in one direction (typically low) although best estimates for the true value of the supersaturation are used in these datasets (*9*, *22*, *23*).

### Synthetic datasets

To evaluate the method of using physics-informed machine learning to discover the functional dependence of ice growth from the mass ratios, we create synthetic datasets with a known depositional growth models. To simulate synthetic data, we start with the observed $S_{i}$, *T*, and $m_{0}$ from the 290 experiments that are shown in fig. S1. Given these initial conditions for $m_{0}$, $S_{i}$, and *T*, we assume that depositional ice growth is described by Eq. 7, with the deposition coefficient function given by Nelson and Baker (*24*) (Eq. 10). We use an ODE solver to integrate Eq. 7 to predict the evolution of the mass ratio for the same length as the experimentally measured time series (up to 500 s). Because the measured mass ratios in the levitation diffusion chamber have high frequency noise, we also use SSA to determine realistic noise for the observed mass ratios. SSA decomposes time series into a sum of temporal principal components that account for a decreasing fraction of the variance in the original time series. Here, we use a window size of 60 s and assume that trailing eigenmodes represent the measurement uncertainty on $\frac{m}{m_{0}}$. An example of the simulated measurements for one time series is given in fig. S3, showing the ODE solution and the SSA reconstruction from the trailing eigenmodes used to estimate measurement uncertainty. All 290 generated synthetic mass time series are shown in fig. S2, which also shows the values for α predicted by Eq. 10 used in generating the synthetic datasets.

### Neural ODE model

For both the strongly and weakly constrained models, we use a fully connected neural network to parameterize the functional dependence in Eqs. 3 and 4 with three linear layers with 50 neurons in each layer, and ReLU activation functions after the first and second layers. Following the third linear layer, we use a sigmoid activation function as both functional forms are expected to be constrained to a range of values (0≤α≤1 and $0<\frac{G}{G_{c}}\leq 2$); without this constraint, it is difficult to optimize the NODE model against observations as predictions can become unphysical. For the NODE, we tested both a fixed step (Runge-Kutta fourth order) and adaptive step size (Runge-Kutta of order 8 of Dormand-Prince-Shampine) as implemented in the torchdiffeq library (*21*) to evaluate how this affects the derived functional dependencies of Eqs. 3 and 4. In cases where the time series are less than 500 s, we mask experimental data such that it is not included in the calculation of the loss function. To minimize the loss function (Eq. 5), we use the AdamW optimizer (*39*), with a base learning rate of 0.01, which we train for 500 epochs, with cosine decay (*40*).

### Symbolic regression

To find a symbolic expression for the function $\alpha =f_{\alpha }\left(S_{i},T|\theta_{\alpha }\right)$ in terms of $S_{i}$ and *T*, we use the binary operators for summation, multiplication, exponentiation, division, and subtraction and the unary operators log, exp, 1/x, square, cube, and tanh, and we run PySR for 1000 generations.

To fit a symbolic expression to $G=G_{c}f_{G}\left(S_{i},T,m|\theta_{G}\right)$, we use the binary operators for summation, multiplication, exponentiation, division, and subtraction and the unary operators 1/x, square, and cube, and we run PySR for 1000 generations. We use *m*, *r*, *T*, $S_{i}$, and $G_{c}$ as input features and target the prediction of *G*; this approach led to more accurate fits to the trained neural network than finding a symbolic expression for the ratio $G/G_{c}$. $G_{c}$ is given by Eq. 12 and does not depend on unknown or unobserved physics, such as the shape of the growing ice crystals.

### AIDA chamber datasets

To evaluate the performance of proposed depositional ice growth models on an independent set of observations, we compare against experiments of cirrus clouds in the AIDA Aerosol and Cloud Chamber. AIDA experiments were taken during the IsoCloud Campaigns, and simulated cirrus clouds between 195 and 235 K and pressures between 150 and 300 hPa, with both heterogeneous and homogeneous ice nuclei. Average ice crystal sizes during these experiments are <10 μm (*16*). We use observations from the Chicago Water Isotope Spectrometer (*33*) of time-evolving vapor pressure of water vapor in the chamber, observations from the APeT instrument (*31*, *41*) measuring after a heated inlet to observe the total water vapor (ice and water vapor) inside the chamber, and measurements of the number concentrations of ice crystals from the welas optical particle counters. In addition, the temperature and pressure of the gas inside the chamber is monitored during experiments. Additional details of the measurements and experiments are given in (*16*, *29*, *30*). To compare against the experimentally observed IWC, we use a bin microphysics model based on the one described in (*16*).

## Supplementary Materials

### This PDF file includes:

Supplementary Text Tables S1 and S2 Figs. S1 to S9 References
