# Supplementary material for: Discovering how ice crystals grow using neural ODEs and symbolic regression
Source: Sci Adv. 2026 Sep 25;12(39):eaee6174. doi: 10.1126/sciadv.aee6174 (PMC13614372; doi:10.1126/sciadv.aee6174)
Supplement: Supplementary file 1 [file sciadv.aee6174_sm.pdf]

Science Advances
AAAS

Supplementary Materials for

Discovering how ice crystals grow using neural ODEs and symbolic regression

Kara D. Lamb *et al.*

Corresponding author: Kara D. Lamb, kl3231@columbia.edu

Sci. Adv. **12**, eaee6174 (2026)
DOI: 10.1126/sciadv.aee6174

This PDF file includes:

Supplementary Text
Tables S1 and S2
Figs. S1 to S9
References

Here we provide addition figures and tables in support of the analysis in the main document.

Constants and Parameterizations used in the Depositional Ice Growth Model

For the depositional ice growth model, we assume that the temperature dependence of saturation vapor pressure with respect to ice (in Pa) is given by (*47*),

$$e_{sat,i}(T) = exp\left(a_0 - \frac{a_1}{T} + a_2 Log(T) - a_3 T\right) \tag{S1}$$

where $a_0 = 9.550426$, $a_1 = 5723.265$, $a_2 = 3.53068$, and $a_3 = 0.00728332$.

The diffusivity of water vapor in air (in m^2/s) is given by

$$D_v = 2.11 \times 10^{-5} \left(\frac{T}{T_0}\right)^{1.94} \left(\frac{p_0}{p}\right) \tag{S2}$$

where $T_0 = 273.15$ K and $p_0 = 101325$ Pa (*12*), which is valid for temperatures between -40 to 40 $°C$.

The thermal conductivity of air in Joules is given by

$$k_a = 4.187 \times 10^{-3}(5.69 + 0.017(T - 273.15)) \tag{S3}$$

Additional constants are given in Table S1.

Table S1: Values of constants used in depositional ice growth model.

Name	Symbol	Value
Density of water	ρ_w	1000 kg/m^3
Density of ice	ρ_i	910 kg/m^3
Molecular mass of water	M_w	18 $\times 10^{-3}$ kg
Thermal deposition coefficient	α_T	1
Latent heat of vaporization	L_v	2.5$\times 10^6$ J/kg
Latent heat of sublimation	L_s	2.837$\times 10^6$ J/kg
Acceleration due to gravity	g	9.81 m/s^2
Universal gas constant	$\mathcal{R}$	8.3144521 J/mole/K
Individual gas constant of air	R_a	287.05 J/kg/K
Individual gas constant of water vapor	R_v	461.51 J/kg/K
Specific heat capacity	c_p	1005
Mean free path of water molecules in air	λ_a	8×10^{-8} m
Vapor jump length	Δ_v	1.3 λ_a
Thermal jump length	Δ_T	2.16×10^{-7} m

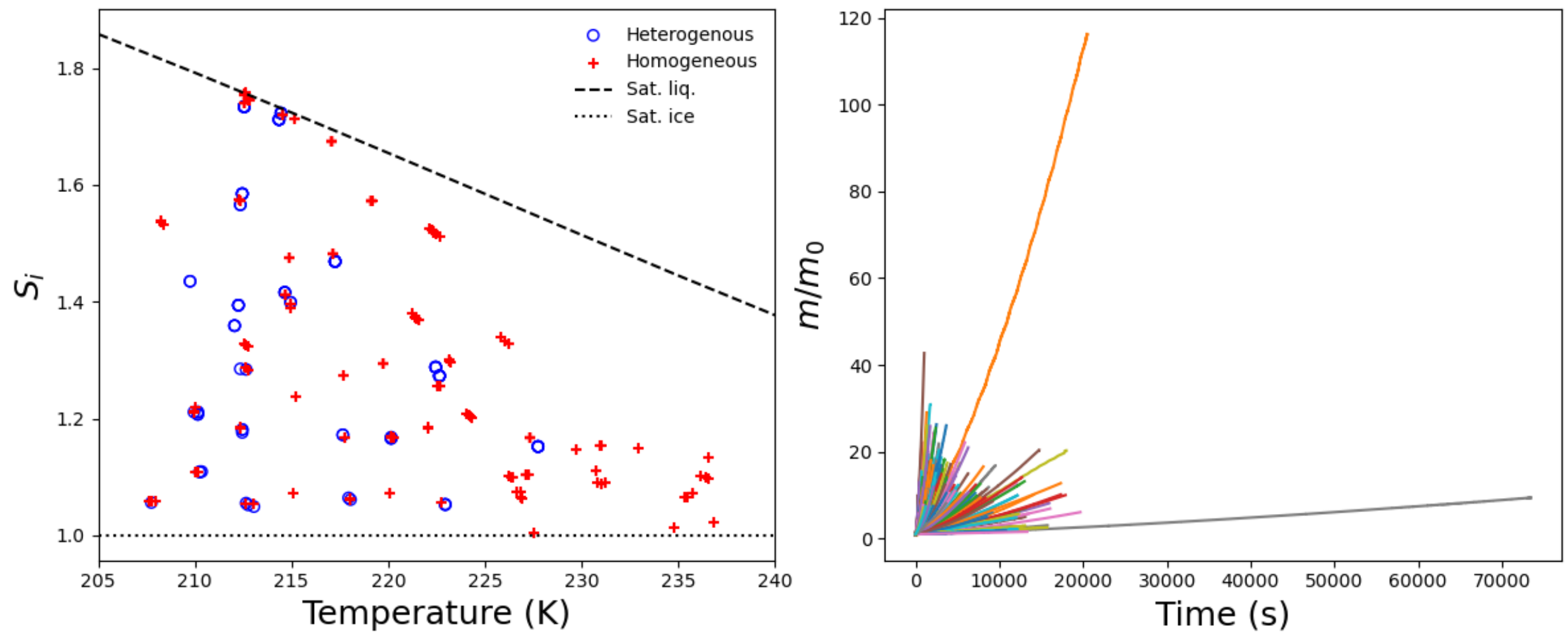

Figure S1: **Overview of experimental data sets used in this analysis. Left:** Saturation with respect to ice and temperature at which experiments included in this analysis were performed. The dashed line shows the temperature dependence of saturation with respect to liquid water, while the dotted line show saturation with respect to ice. Symbols indicate whether ice crystals were nucleated heterogeneously or homogeneously. **Right:** Mass ratios of all ice crystals as a function of time.

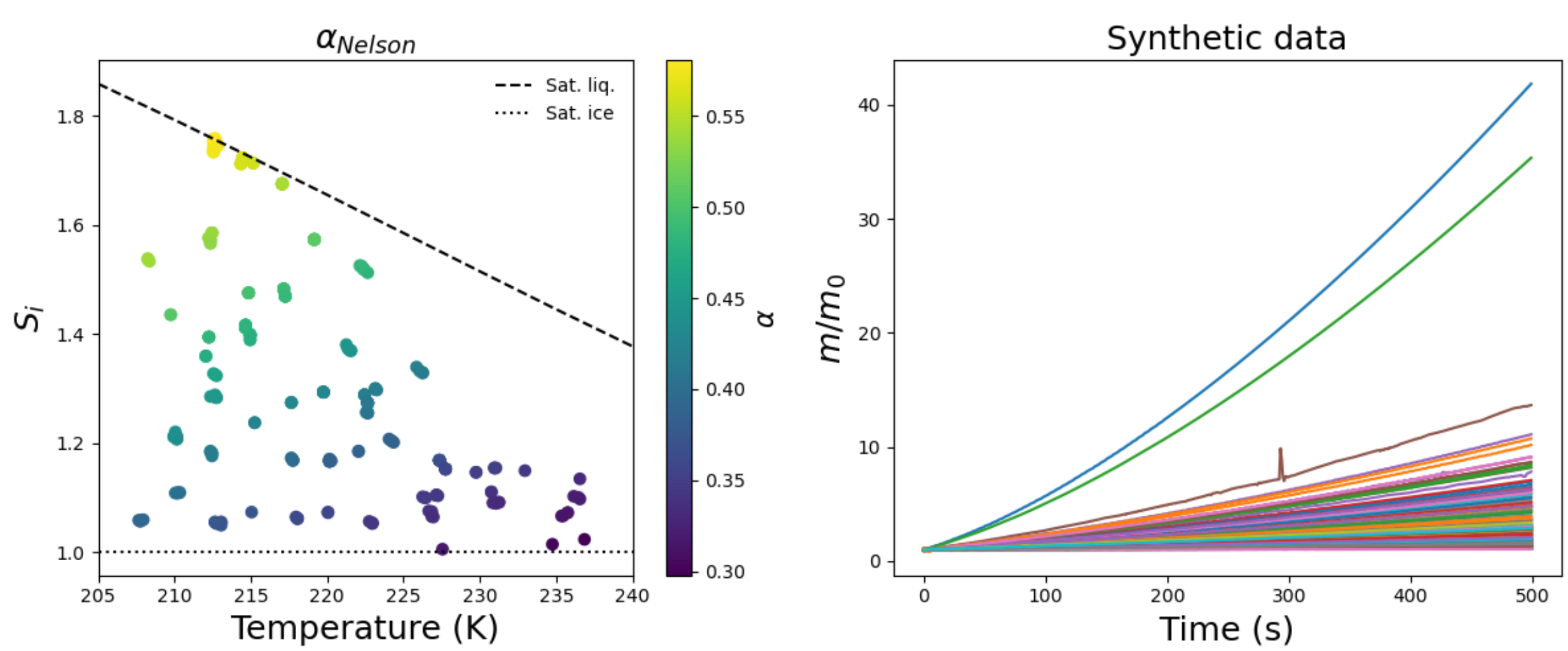

Figure S2: **Synthetic data sets. Left:** Values for α predicted by Nelson and Baker, 1996 (*24,*) parameterization for the S_i and T conditions for the 290 experimental data sets (used as initial conditions in creating the synthetic mass time series). **Right:** Times series of mass ratios for all of the synthetic data.

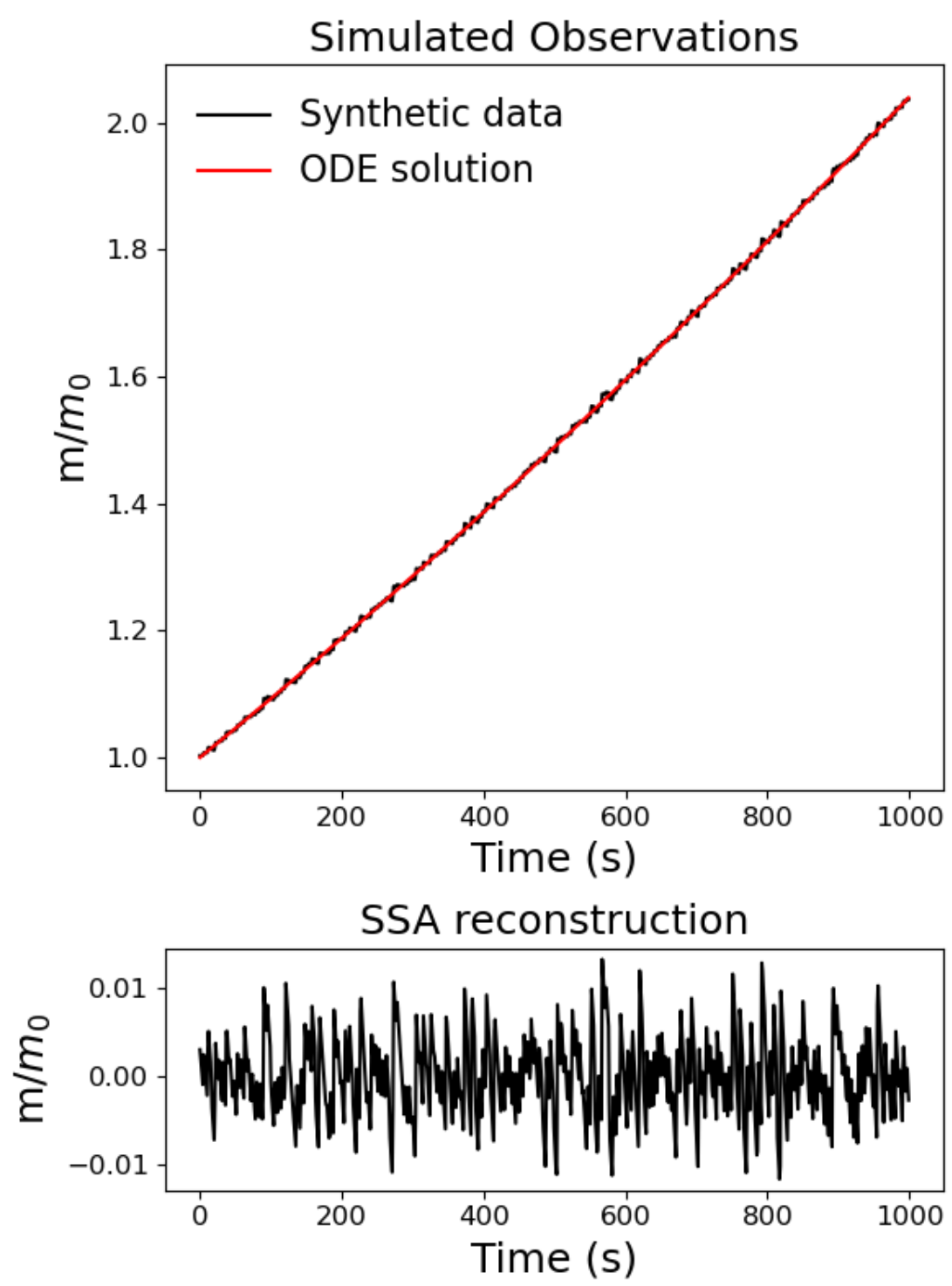

Figure S3: **Example of a synthetic time series of the ice mass ratio.** Measurement uncertainty is derived from the trailing eigenmodes of a singular spectrum analysis of the observed time series.

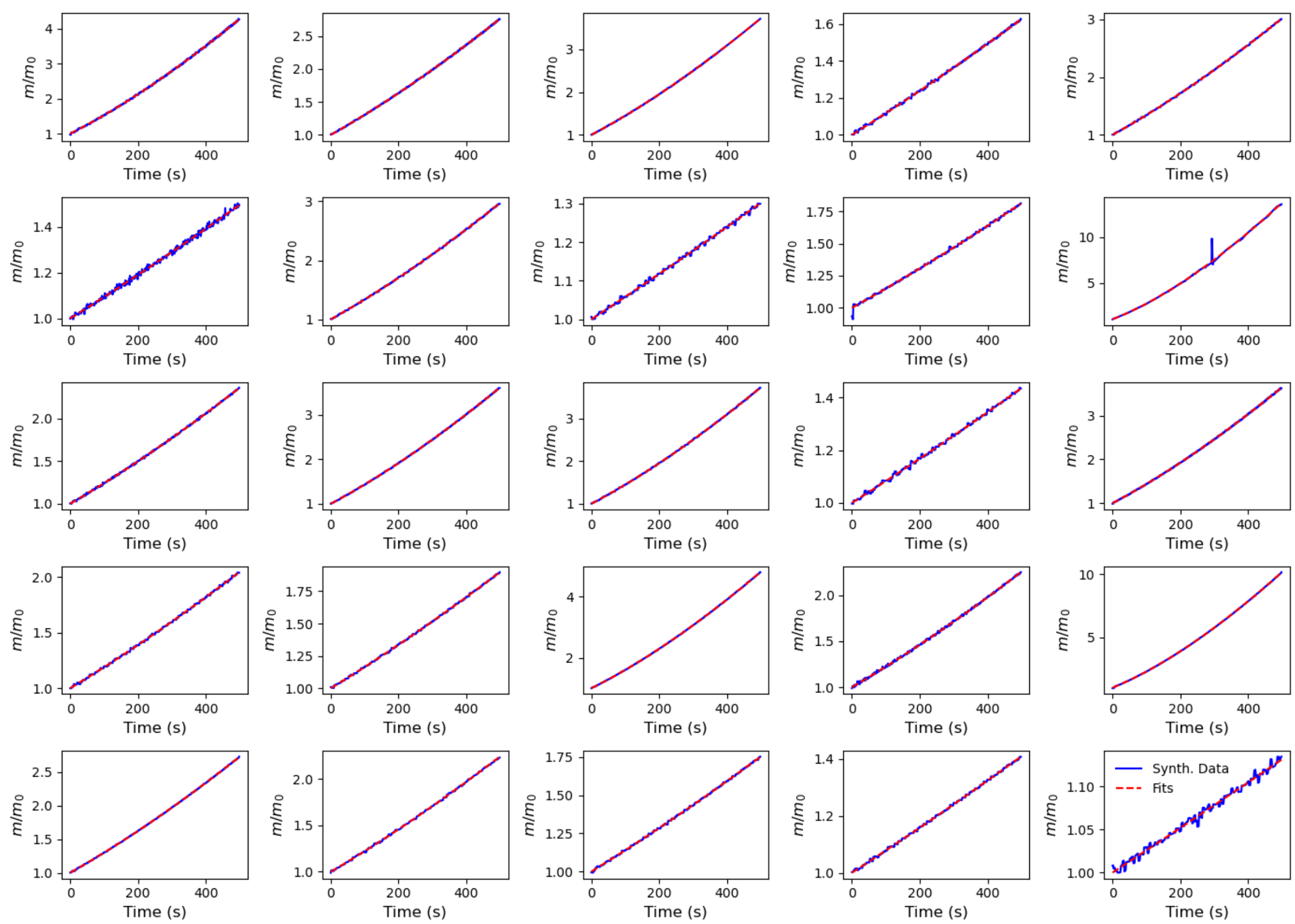

Figure S4: **Model predictions for individual experiments for the synthetic data sets.** Synthetic mass ratios and strongly-constrained NODE model predictions for a subset of the time series.

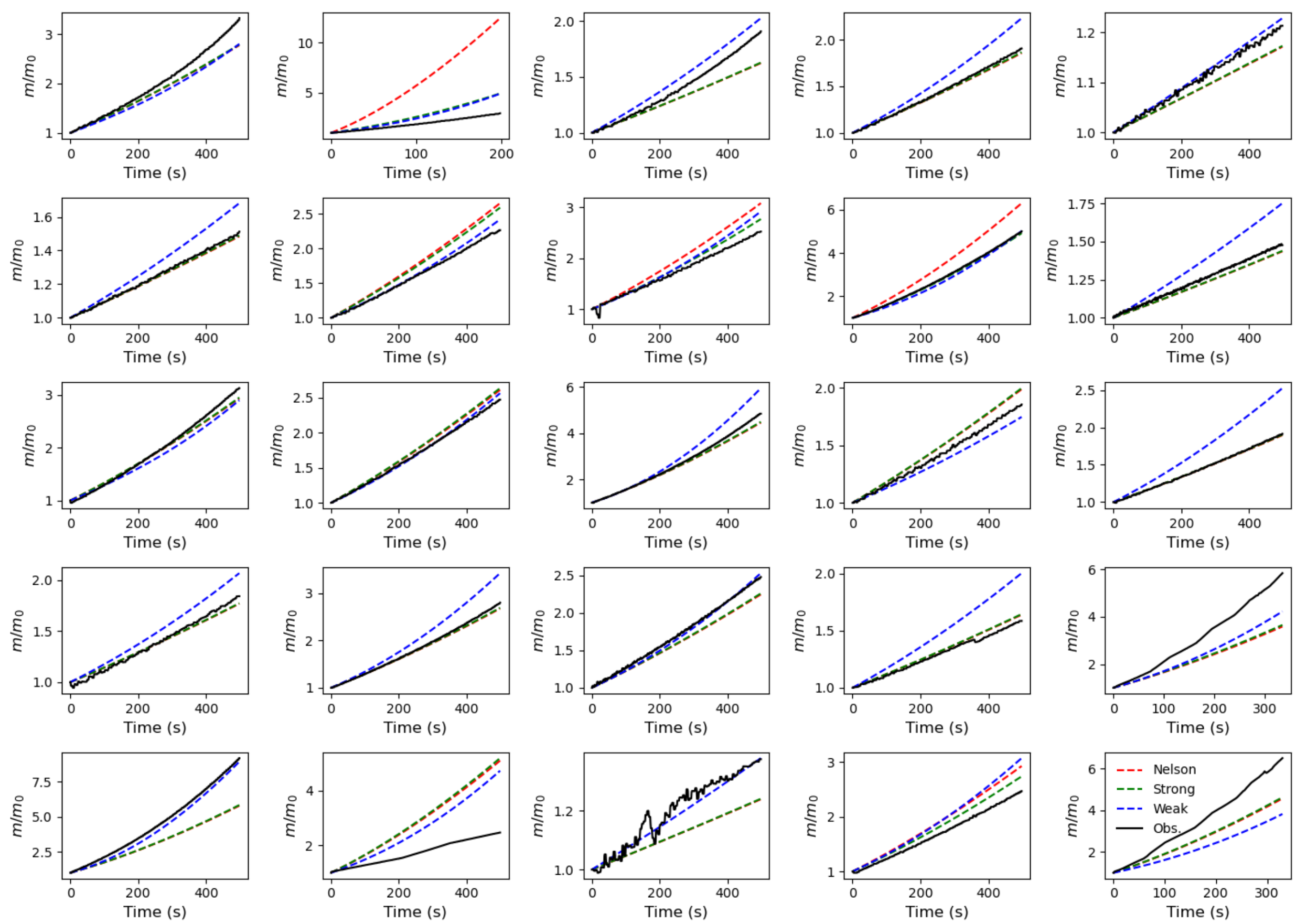

Figure S5: **Model predictions for individual experiments for the real data sets.** Nelson and Baker model predictions, strongly-constrained NODE model predictions, and weakly-constrained NODE model predictions compared to observations for a subset of the time series.

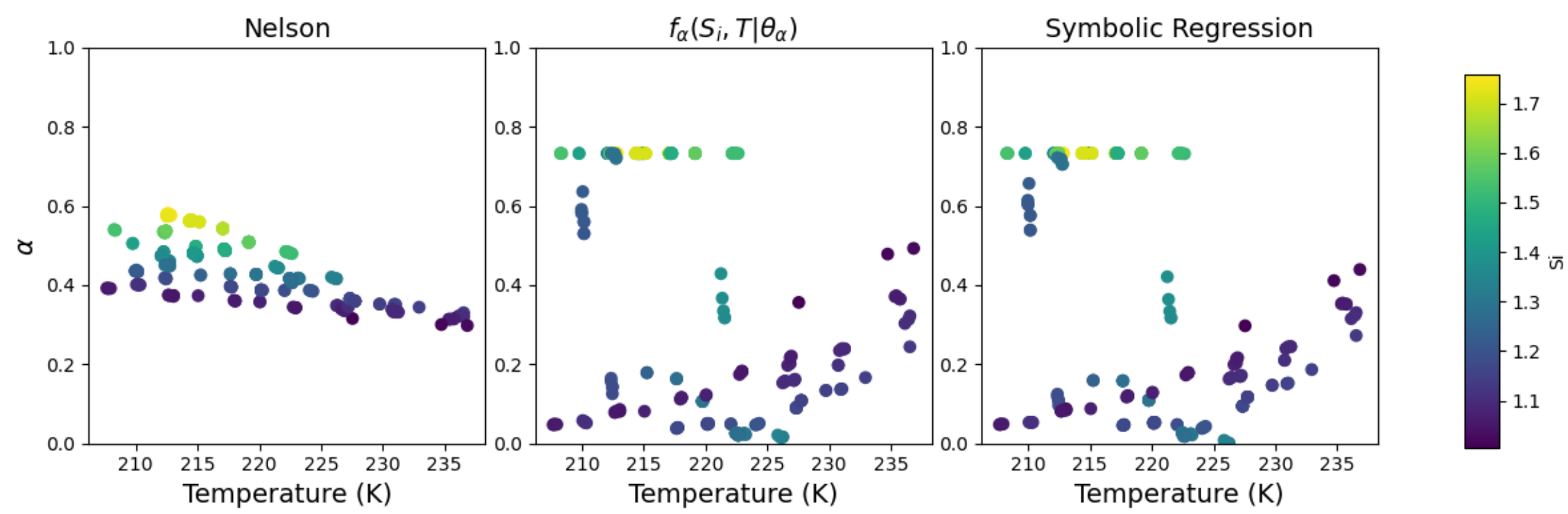

Figure S6: **Functional dependence of α learned from the real data.** a) Saturation and temperature dependence of α parameterization from Nelson and Baker, 1996 (*24,*). b) Saturation and temperature dependence from the trained NN for the real data sets. c) Predictions from an expression learned by symbolic regression from the trained NN for the real data sets.

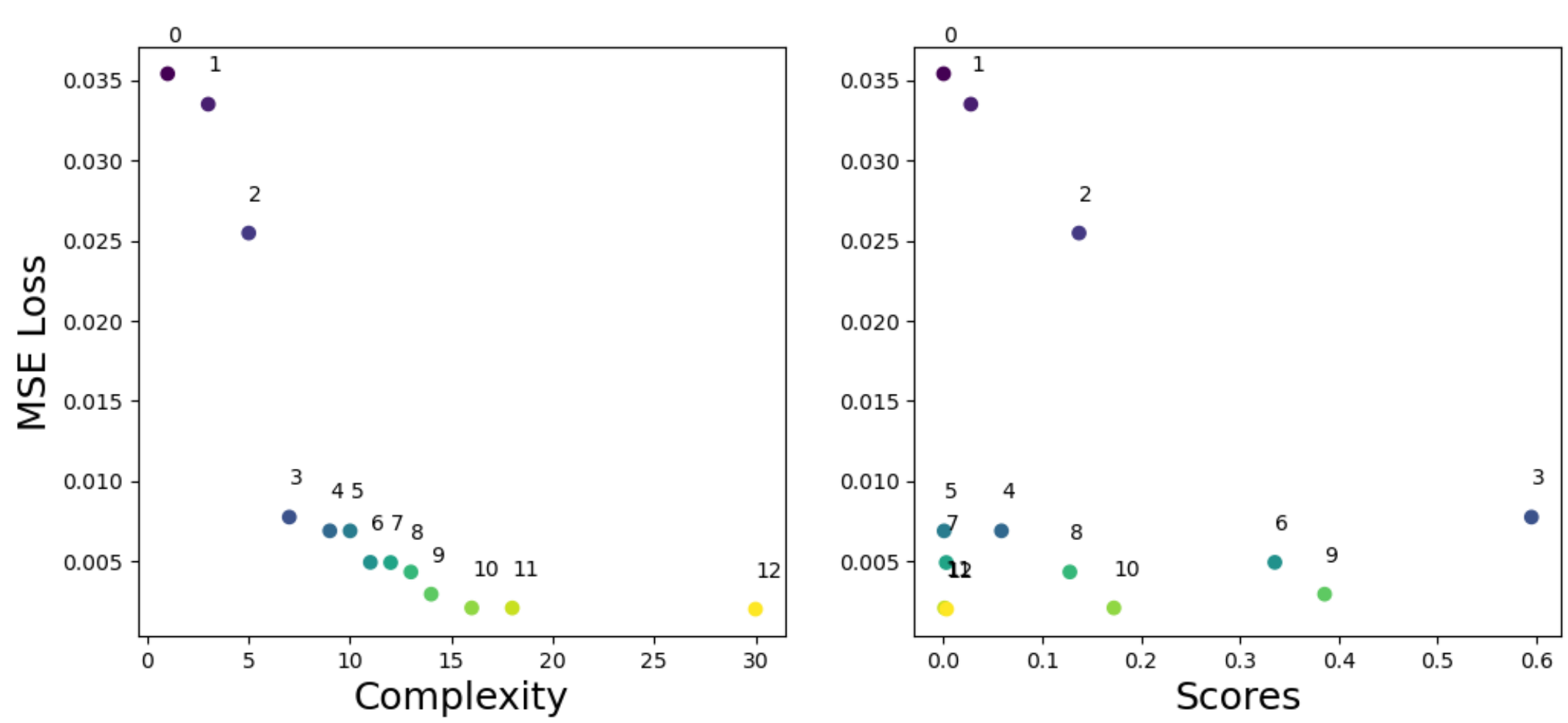

Figure S7: **Comparison of MSE loss (after 500 seconds) for each symbolic regression expression.** Equation numbers correspond to the rows in Table S2. The complexity of the equation is defined as the number of a nodes in an expression tree. The score is defined as the negative of the log-loss with respect to complexity.

Table S2: Equations for G discovered by symbolic regression.

#	**Discovered Equation**
0	G_c
1	$0.93458 \times G_c$
2	$G_c - 0.347 \times 10^{-21} m^{-1}$
3	$652.8 \times G_c m^{0.253}$
4	$\frac{G_c}{3.50179 \times 10^{-6} m^{-0.469} + 0.413}$
5	$\frac{G_c}{2.89192 \times 10^{-6} m^{-0.476} + 0.419}$
6	$G_c \left[0.615 + \frac{3.13 \times 10^{-9}}{0.836 G_c + 1000m}\right]^{-1}$
7	$G_c \left[0.615 + \frac{3.13 \times 10^{-9}}{0.902 G_c + 1000m}\right]^{-1}$
8	$688.267 G_c^{1.3153} \left[0.85601 + \frac{2.6606 \times 10^{-12}}{m}\right]^{-1} + 0.1123 \times 10^{-9}$
9	$\left[\left(0.803 + 2.16 \times 10^{-12} m^{-1}\right)\left(2.7649 \times 10^{-7} G_c^{-0.743} - 0.360\right)\right]^{-1} \times 10^{-9}$
10	$\left[\left(0.504 + \frac{2.86}{m \times 10^{12} + 2.08}\right)\left(1.2287^{-7} G_c^{-0.795} - 0.390\right)\right]^{-1} \times 10^{-9}$
11	$\left[\left(1.1805 \times 10^{-7} G_c^{-0.797} - 0.389\right)\left(0.5018 + \frac{2.85}{m \times 10^{12} + 2.06}\right)\right]^{-1} \times 10^{-9}$
12	$\left[\left(0.591 + \frac{2.83}{\frac{m \times 10^{12} + 0.726}{0.736} + 0.224}\right)\left(\left(\frac{2.01}{G_c \times 10^9}\right)^{0.799} - 0.409\right) - 0.355 + \frac{0.340}{\left(\frac{1.61}{G_c \times 10^9}\right)^{(S_i - 1)}}\right]^{-1} \times 10^{-9}$

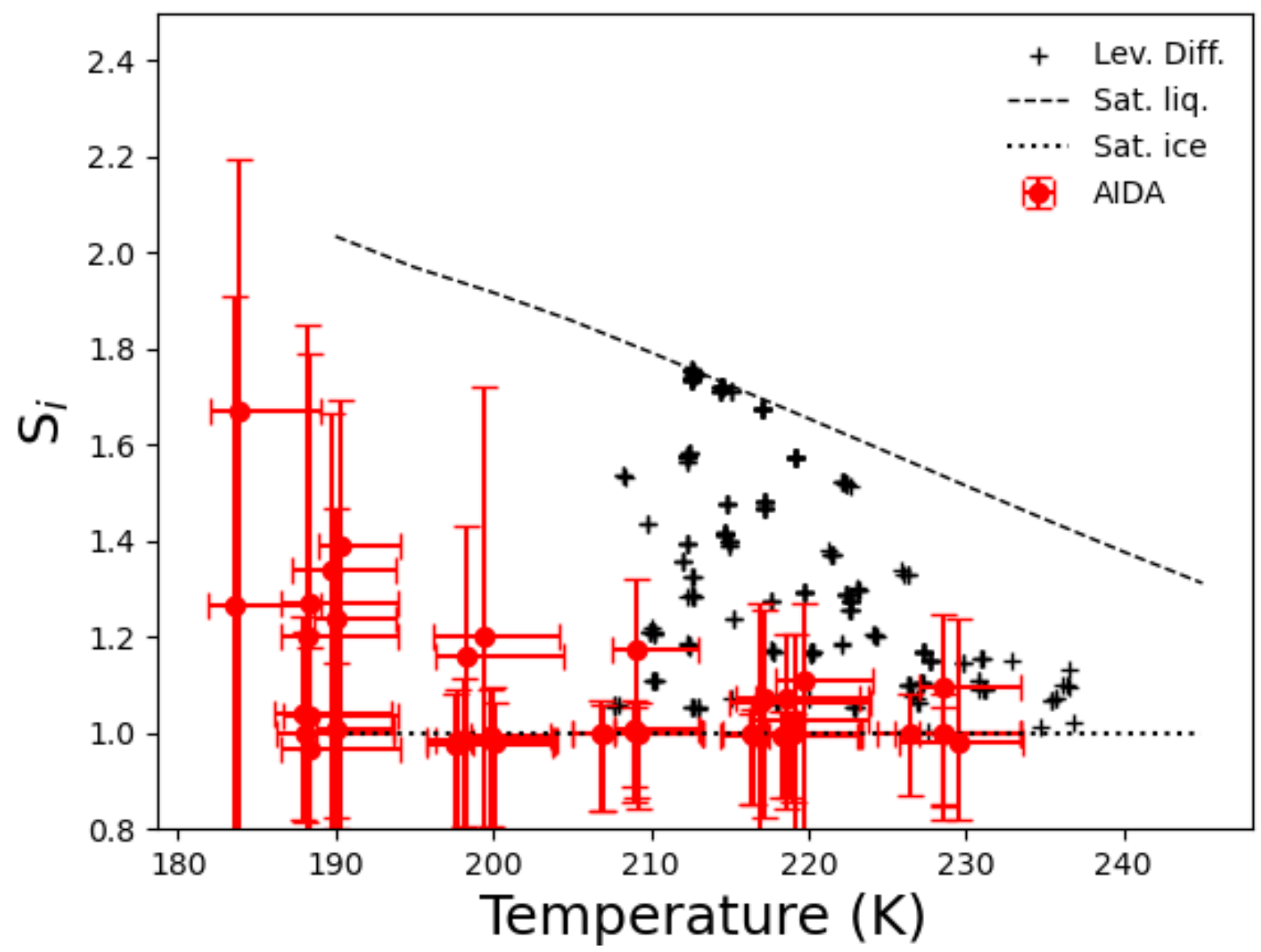

Figure S8: **Temperature and Supersaturation range for AIDA experiments compared with levitation diffusion chamber.**

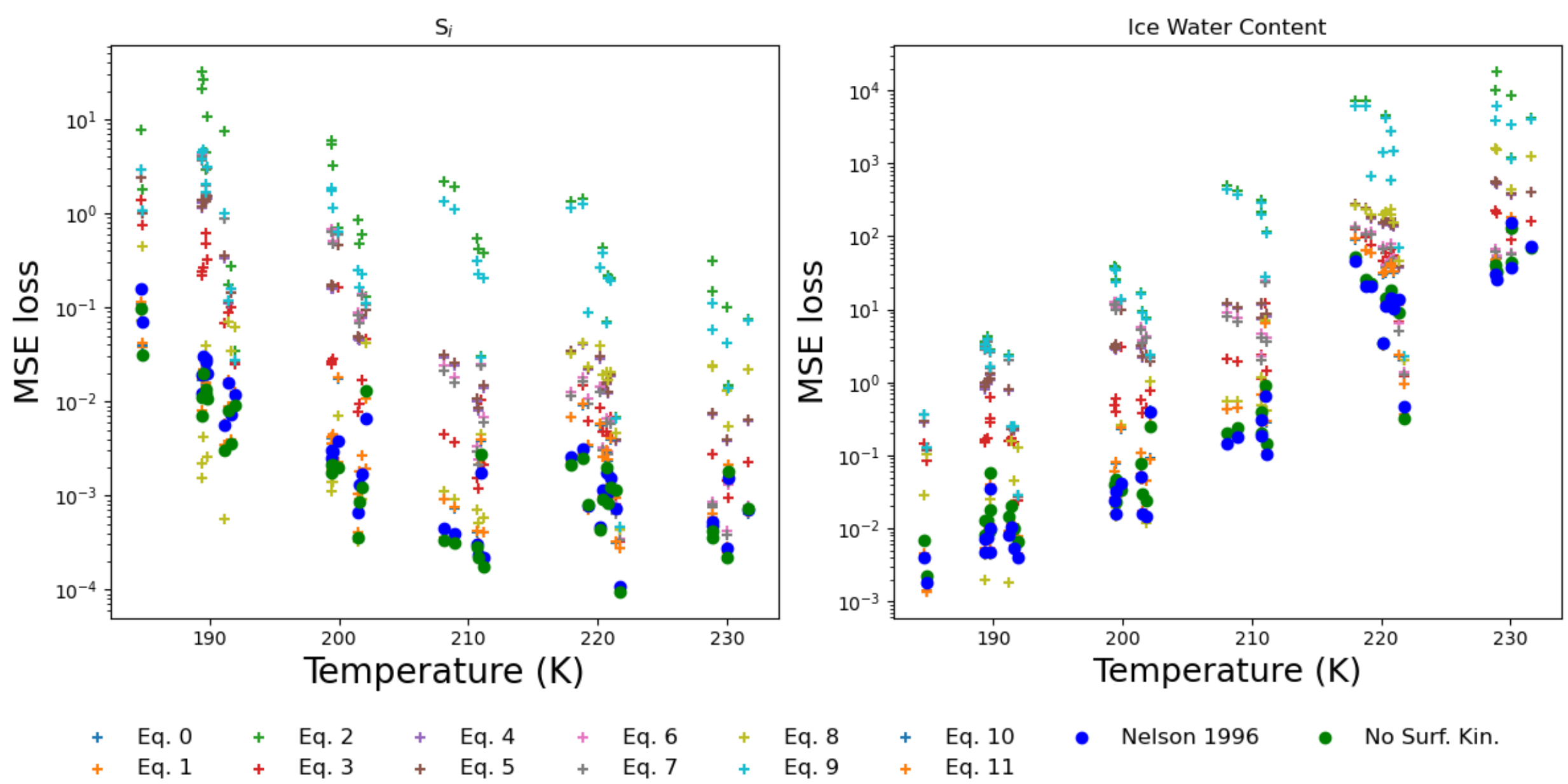

Figure S9: **Comparison between AIDA experiments and bin microphysics model**. MSE loss for S_i (left) and IWC (right) between bin microphysics model using discovered equations for G given in Table S2 and observations from AIDA.

REFERENCES

1. B. Gasparini, S. C. Sullivan, A. B. Sokol, B. Kärcher, E. Jensen, D. L. Hartmann, Opinion: Tropical cirrus—From micro-scale processes to climate-scale impacts. *Atmos. Chem. Phys.* **23**, 15413–15444 (2023).

2. S. Solomon, K. H. Rosenlof, R. W. Portmann, J. S. Daniel, S. M. Davis, T. J. Sanford, G.-K. Plattner, Contributions of stratospheric water vapor to decadal changes in the rate of global warming. *Science* **327**, 1219–1223 (2010).

3. B. Kärcher, Formation and radiative forcing of contrail cirrus. *Nat. Commun.* **9**, 1824 (2018).

4. S. C. Sullivan, A. Voigt, Ice microphysical processes exert a strong control on the simulated radiative energy budget in the tropics. *Commun. Earth Environ.* **2**, 137 (2021).

5. F. Lamraoui, M. Krämer, A. Afchine, A. B. Sokol, S. Khaykin, A. Pandey, Z. Kuang, Sensitivity of convectively driven tropical tropopause cirrus properties to ice habits in high-resolution simulations. *Atmos. Chem. Phys.* **23**, 2393–2419 (2023).

6. A. Avramov, J. Y. Harrington, Influence of parameterized ice habit on simulated mixed phase Arctic clouds. *J. Geophys. Res. Atmos.* **115**, D03205 (2010).

7. T. Gonda, T. Yamazaki, Morphology of ice droxtals grown from supercooled water droplets. *J. Cryst. Growth* **45**, 66–69 (1978).

8. T. Gonda, T. Yamazaki, Initial growth forms of snow crystals growing from frozen cloud droplets. *J. Meteorol. Soc. Jpn.* **62**, 190–192 (1984).

9. G. F. Pokrifka, A. M. Moyle, L. E. Hanson, J. Y. Harrington, Estimating surface attachment kinetic and growth transition influences on vapor-grown ice crystals. *J. Atmos. Sci.* **77**, 2393–2410 (2020).

10. D. Rui, G. F. Pokrifka, A. M. Moyle, J. Y. Harrington, The growth of ice crystals formed from frozen solution droplets: Laboratory measurements and power-law parameterization. *J. Atmos. Sci.* **82**, 775–788 (2025).

11. H. Morrison, M. van Lier Walqui, A. M. Fridlind, W. W. Grabowski, J. Y. Harrington, C. Hoose, A. Korolev, M. R. Kumjian, J. A. Milbrandt, H. Pawlowska, D. J. Posselt, O. P. Prat, K. J. Reimel, S.-I. Shima, B. van Diedenhoven, L. Xue, Confronting the challenge of modeling cloud and precipitation microphysics. *J. Adv. Model. Earth Syst.* **12**, e2019MS001689 (2020).

12. H. R. Pruppacher, J. D. Klett, *Microphysics of Clouds and Precipitation* (Kluwer Academic Publishers, ed. 2, 1998).

13. N. Magee, A. M. Moyle, D. Lamb, Experimental determination of the deposition coefficient of small cirrus-like ice crystals near −50°C. *Geophys. Res. Lett.* **33**, L17813 (2006).

14. J. Skrotzki, P. Connolly, M. Schnaiter, H. Saathoff, O. Möhler, R. Wagner, M. Niemand, V. Ebert, T. Leisner, The accommodation coefficient of water molecules on ice–cirrus cloud studies at the AIDA simulation chamber. *Atmos. Chem. Phys.* **13**, 4451–4466 (2013).

15. J. Y. Harrington, A. Moyle, L. E. Hanson, H. Morrison, On calculating deposition coefficients and aspect-ratio evolution in approximate models of ice crystal vapor growth. *J. Atmos. Sci.* **76**, 1609–1625 (2019).

16. K. D. Lamb, J. Y. Harrington, B. W. Clouser, E. J. Moyer, L. Sarkozy, V. Ebert, O. Möhler, H. Saathoff, Re-evaluating cloud chamber constraints on depositional ice growth in cirrus clouds—Part 1: Model description and sensitivity tests. *Atmos. Chem. Phys.* **23**, 6043–6064 (2023).

17. M. Schmidt, H. Lipson, Distilling free-form natural laws from experimental data. *Science* **324**, 81–85 (2009).

18. M. Cranmer, A. Sanchez Gonzalez, P. Battaglia, R. Xu, K. Cranmer, D. Spergel, S. Ho, Discovering symbolic models from deep learning with inductive biases. *Adv. Neural Inf. Proces. Syst.* **33**, 17429–17442 (2020).

19. S.-M. Udrescu, M. Tegmark, AI Feynman: A physics-inspired method for symbolic regression. *Sci. Adv.* **6**, eaay2631 (2020).

20. M. Raissi, P. Perdikaris, G. E. Karniadakis, Physics-informed neural networks: A deep learning framework for solving forward and inverse problems involving nonlinear partial differential equations. *J. Comput. Phys.* **378**, 686–707 (2019).

21. R. T. Chen, Y. Rubanova, J. Bettencourt, D. K. Duvenaud, Neural ordinary differential equations. *Adv. Neural Inf. Proces. Syst.* **31**, 6571–6583 (2018).

22. A. Harrison, A. M. Moyle, M. Hanson, J. Y. Harrington, Levitation diffusion chamber measurements of the mass growth of small ice crystals from vapor. *J. Atmos. Sci.* **73**, 2743–2758 (2016).

23. G. F. Pokrifka, A. M. Moyle, J. Y. Harrington, Effective density derived from laboratory measurements of the vapor growth rates of small ice crystals at- 65 to- 40 C. *J. Atmos. Sci.* **80**, 501–517 (2023).

24. J. T. Nelson, M. B. Baker, New theoretical framework for studies of vapor growth and sublimation of small ice crystals in the atmosphere. *J. Geophys. Res. Atmos.* **101**, 7033–7047 (1996).

25. K. M. Gierens, M. Monier, J.-F. Gayet, The deposition coefficient and its role for cirrus clouds. *J. Geophys. Res. Atmos.* **108**, 4069 (2003).

26. J. Y. Harrington, G. F. Pokrifka, Approximate models for lateral growth on ice crystal surfaces during vapor depositional growth. *J. Atmos. Sci.* **78**, 967–981 (2021).

27. J. Y. Harrington, D. Lamb, R. Carver, Parameterization of surface kinetic effects for bulk microphysical models: Influences on simulated cirrus dynamics and structure. *J. Geophys. Res. Atmos.* **114**, D06212 (2009).

28. M. Cranmer, Interpretable machine learning for science with PySR and SymbolicRegression.jl. arXiv:2305.01582 [astro-ph.IM] (2023).

29. K. D. Lamb, B. W. Clouser, M. Bolot, L. Sarkozy, V. Ebert, H. Saathoff, O. Möhler, E. J. Moyer, Laboratory measurements of HDO/H_2O isotopic fractionation during ice deposition in simulated cirrus clouds. *Proc. Natl. Acad. Sci. U.S.A.* **114**, 5612–5617 (2017).

30. B. W. Clouser, K. D. Lamb, L. C. Sarkozy, J. Habig, V. Ebert, H. Saathoff, O. Möhler, E. J. Moyer, No anomalous supersaturation in ultracold cirrus laboratory experiments. *Atmos. Chem. Phys.* **20**, 1089–1103 (2020).

31. J. Skrotzki, "High-accuracy multiphase humidity measurements using TDLAS: Application to the investigation of ice growth in simulated cirrus clouds," thesis, University of Heidelberg (2012).

32. D. W. Fahey, R. S. Gao, O. Möhler, H. Saathoff, C. Schiller, V. Ebert, M. Krämer, T. Peter, N. Amarouche, L. M. Avallone, R. Bauer, Z. Bozoki, L. E. Christensen, S. M. Davis, G. Durry, C. Dyroff, R. L. Herman, S. Hunsmann, S. M. Khaykin, P. Mackrodt, J. Meyer, J. B. Smith, N. Spelten, R. F. Troy, H. Vömel, S. Wagner, F. G. Wienhold, The AquaVIT-1 intercomparison of atmospheric water vapor measurement techniques. *Atmos. Meas. Tech.* **7**, 3177–3213 (2014).

33. L. C. Sarkozy, B. W. Clouser, K. D. Lamb, E. J. Stutz, H. Saathoff, O. Möhler, V. Ebert, E. J. Moyer, The Chicago Water Isotope Spectrometer (ChiWIS-lab): A tunable diode laser spectrometer for chamber-based measurements of water vapor isotopic evolution during cirrus formation. *Rev. Sci. Instrum.* **91**, 045120 (2020).

34. W. Mordy, Computations of the growth by condensation of a population of cloud droplets. *Tellus* **11**, 16–44 (1959).

35. M. L. Duffy, B. Medeiros, A. Gettelman, T. Eidhammer, Perturbing parameters to understand cloud contributions to climate change. *J. Climate* **37**, 213–227 (2024).

36. K. D. Lamb, C. E. Singer, K. Loftus, H. Morrison, M. Powell, J. Ko, J. Buch, A. Z. Hu, M. van Lier Walqui, P. Gentine, Perspectives on systematic cloud microphysics scheme development with machine learning. *J. Adv. Model. Earth Syst.* **18**, e2025MS005341 (2026).

37. D. Lamb, J. Verlinde, *Physics and Chemistry of Clouds* (Cambridge Univ. Press, 2011).

38. N. J. Bacon, M. B. Baker, B. D. Swanson, Initial stages in the morphological evolution of vapour-grown ice crystals: A laboratory investigation. *Q. J. Roy. Meteorol. Soc.* **129**, 1903–1927 (2003).

39. I. Loshchilov, F. Hutter, Decoupled weight decay regularization. arXiv:1711.05101 [cs.LG] (2017).

40. I. Loshchilov, F. Hutter, SGDR: Stochastic gradient descent with warm restarts. arXiv:1608.03983 [cs.LG] (2016).

41. V. Ebert, C. Lauer, H. Saathoff, S. Hunsmann, S. Wagner, Simultaneous, absolute gas-phase and total water detection during cloud formation studies in the AIDA chamber using a dual 1.37 μm TDL-Spectrometer. *Geophys. Res. Abstr.* **10**, 1–2 (2008).

42. K. D. Lamb, Code for "Discovering how ice crystals grow using neural ODE's and symbolic regression," version v.1.0.0, Zenodo (2026); https://zenodo.org/records/20468474.

43. A. Harrison, A. Moyle, M. Hansen, J. Y. Harrington, Electrodynamic levitation diffusion chamber measurements of the mass growth of heterogeneously-nucleated ice crystals grown from the vapor, Data Commons (2016); www.datacommons.psu.edu/commonswizard/MetadataDisplay.aspx?Dataset=6184.

44. G. Pokrifka, A. Moyle, J. Y. Harrington, Electrodynamic levitation diffusion chamber measurements of the mass growth of homogeneously-nucleated ice crystals grown from the vapor, Data Commons (2018); www.datacommons.psu.edu/commonswizard/MetadataDisplay.aspx?Dataset=6185.

45. G. Pokrifka, A. Moyle, J. Y. Harrington, Electrodynamic levitation diffusion chamber measurements of the mass growth of ice crystals grown from the vapor at −65 to −40C, Data Commons (2022); www.datacommons.psu.edu/commonswizard/MetadataDisplay.aspx?Dataset=6339.

46. K. D. Lamb, J. Y. Harrington, B. W. Clouser, E. J. Moyer, L. Sarkozy, V. Ebert, O. Möhler, H. Saathoff, IsoCloud4 data sets for depositional ice growth analysis, Data Commons (2023); https://zenodo.org/records/7986868.

47. D. M. Murphy, T. Koop, Review of the vapour pressures of ice and supercooled water for atmospheric applications. *Q. J. Roy. Meteorol. Soc.* **131**, 1539–1565 (2005).
